# Effects of Different Types of Physical Activity on Health-Related Quality-of-Life in Korean Women with Depressive Disorder

**DOI:** 10.3390/ijerph18094639

**Published:** 2021-04-27

**Authors:** Kyoman Koo, Kyungjin Kim

**Affiliations:** 1 Department of Adapted Physical Education, Baekseok University, Cheonan-si 31065, Korea; nicekm73@hanmail.net; 2 Department of Adapted Physical Activity, Korea Nazarene University, Cheonan-si 31172, Korea

**Keywords:** physical activity, health related quality of life, Korean women, depressive disorder

## Abstract

Depressive disorder is a frequent psychological illness and causes community health problems for many women. It was found that the health-related quality of life (HRQoL) score of many women was altered due to mental and physical problems. Physical activity (PA) might effectively improve the responses of the HRQoL of women with depressive disorder. Therefore, the study aimed to identify the effects of different types of PA (e.g., walking, strength exercise, flexibility exercise) on the responses of the HRQoL of Korean women with depressive disorder. A sample of 1315 Korean women aged 19 or older with a depressive disorder was accumulated. The Korea National Health and Nutrition Examination Survey (KNHANES) and Euro Quality of Life-5 Dimensions (EQ-5D index score) were used. The characteristics of the participants were analyzed by the complex sample in frequency analysis. Furthermore, the complex sample general linear model was used to determine the effects of different types of PA on the HRQoL of Korean women with depressive disorder. In the results, there was a statistically significant difference between the groups who participated for one to two days, who participated for three to four days, and who did not participate in walking at all. In the flexibility exercise, there was a statistically significant difference in the group who participated for three to four days from the group who did not participate at all. However, there was no statistically significant difference in the strength exercise. In conclusion, the walking and flexibility exercises were effective physical activities (PAs) to improve the responses to the HRQoL of Korean women with depressive disorder.

## 1. Introduction

Depressive disorder is a frequent psychological illness and a growing community health problem in the world. It was anticipated to be a common public health problem after 2020 [1]. For instance, it was estimated that 7.1% of all American people (17.3 million adults aged 18 or older in 2017) had a major depression symptom [2]. An estimated 19.7% of UK individuals aged 16 or above had a depression episode and a 1.5% raise every year before 2014 [3]. According to the World Health Organization (WHO), more than 264 million individuals were influenced by depressive episodes in 2020 [4]. Depression can lead the affected people to function poorly in diverse places, such as at home, school, and the workplace. The WHO recommends that people with depressive disorder should be considered in need of care. Especially, it can cause a serious health problem when long-lasting with moderate to severe intensity. In serious cases, it can lead to suicide [5].

Depressive disorder is more common in women than men across the world. The occurrence of a major depressive symptom was higher in adult women (8.7%) than adult men (5.3%) in the United States [2]. Women experience various changes when getting older and face big issues in life, such as pregnancy, childbearing, and menopause [6]. For example, middle-aged women can have physical health problems due to physiological changes, such as menopause [7]. The situation of having to take on various roles and responsibilities, such as childbearing and parenting, can cause mental and psychological health problems of women, such as depressive disorder. Furthermore, the responses to the health-related quality of life (HRQoL) of many women are affected by depression, anxiety, self-esteem, aging, income level, health, and stress [8]. According to the WHO [4], the HRQoL can be defined as a concept of multiple dimensions, including physical, emotional, social, and mental function. It is related to population health, causes of death, life expectancy, and health status on quality of life [9]. Moreover, depressive disorder has a major impact on the responses to the HRQoL, as reported literature indicated. These factors, including pregnancy, childbearing, menopause, and aging, provide adult women with stress, which causes depression [10,11].

Physical activity (PA) can reduce and prevent depressive disorder in women. Several studies provide evidence that the mental health of adult women was positively influenced by PA [8,12,13,14,15]. Mourady et al. [16] reported that the quality of life might correlate with PA and depression, and Pereira et al. [17] stated that the PA helped reduce scores of depression symptoms in women during and after pregnancy. According to Robledo-Colonia et al. [18], aerobic exercise reduces depressive episodes in pregnant women. Jung et al. [19] reported that aqua aerobics exercise reduces depression and improves the quality of life among climacteric women. Additionally, PA is effective for reducing and preventing stress, which affects mental health positively [20,21]. Therefore, the PA can assist in improving the HRQoL of women with depressive disorder.

In South Korea, depression disorder is a common mental disease among Korean women. The Korea Ministry of Health and Welfare [22,23] reported that about 25% of adult Korean women suffer from depression symptoms and mental illness in their lives, and depression is twice more common in Korean adult women than adult men. Many Korean women are under more stress than Korean men because they have more social pressure (e.g., low economic level, low social position, pregnancy, childbirth, childbearing, menopause) than Korean men [24]. These conditions can lead many Korean women to have mental issues or depression episodes. The depression disorder of Korean women should be managed because their level of interest and activity in daily life can be decreased, leading to an isolated life and possibly suicide [25]. Thus, if it is not properly administered, the HRQoL will inevitably deteriorate. In addition, PA might be the best way to overcome the depressive disorder of women.

Although PA has a significant association with depressive disorder and HRQoL of women, there are not many studies to examine different age groups and different types of PA. Some intervention studies for mental diseases reported the effects of PA on the quality of life or depressive disorder in only pregnant women [16,17,18]. Several researchers determined how PA reduces and prevents depressive symptoms for only young age women, climacteric women, or elderly women [19,20,21]. Furthermore, many researchers investigated the effect of only one type of PA on depressive disorder or HRQoL rather than different types of PA [26,27,28]. This limited evidence cannot provide individuals who have depressive disorder or interests in mental illness with a variety of information and the knowledge of which PA is good for them. This study used the Korea National Health and Nutrition Examination Survey (KNHANES) to determine the effects of PA on the HRQoL of Korean women with depressive disorder because it can resolve the restrictions about the number of participants and different types of PA. Therefore, the aim of this study was to determine the effects of different types of PA (e.g., walking, strength exercise, flexibility exercise) on the HRQoL of Korean women with depressive disorder. In addition, the hypothesis of the study was that there are significant differences between the effects of different types of PA on the HRQoL of Korean women with depressive disorder.

## 2. Materials and Methods

### 2.1. Participants

The aim of this study was to identify the effects of different types of PA (e.g., walking, strength exercise, flexibility exercise) on the HRQoL of Korean women with depression disorder, using raw data from the KNHANES. The KNHANES was conducted by the recommendations of the WHO and with the approval of the institutional review board in Korea Centers for Disease Control and Prevention. Furthermore, the statistical data of the KNHANES are representative of South Korea, which is carried out annually after sampling by the laminar collection system extraction method. In this study, the 4th, 5th, and 6th data of the KNHANES from 2007–2015 were vertically merged and analyzed to increase statistical power. The 4th data were conducted in 2007–2009, in which 24,871 people participated in 500 survey districts nationwide, and the 5th data were conducted in 2010–2012, with 31,596 people participating in 576 survey districts nationwide. The 6th data were conducted in 2013–2015, in which 22,948 people participated in 576 survey districts nationwide. In this study, data of a total of 1315 (unweighted cases) participants who were over 19 years of age and who responded with current depression as to the prevalence of depression were used for analysis. The demographic characteristics of participants are shown in Table 1.

### 2.2. Research Instrument

The International Physical Activity Questionnaire (IPAQ) was standardized to measure the level of PA of people aged 16–65 throughout the world, which helped establish the KNHANES. The IPAQ has a long-form and short-form questionnaire and was considered culturally diverse [29]. Craig et al. [30] provided the validation (e.g., Pearson correlation coefficient ranging from 1.12–0.57) of the short form of the IPAQ. The KNHANES is a short form questionnaire derived from the part of the IPAQ that is meant to accumulate data determining the health score of Koreans. The sampling frame of the KNHANES used the data from the latest Population and Housing Census available at the time of sampling design. Through this process, the representative samples were collected from South Korean citizens aged one year or older, which was the target population. The KNHANES consists of a household member confirmation survey, a health survey, a medical examination survey, and a nutrition survey. For this study, only the health survey was used, which was determined by the purpose of the study. The household member verification survey is a basic survey for conducting KNHANES. It is a survey that identifies the status of all dwellings and households in the selected area through sample design and selects households to participate in health surveys and examination surveys. The health survey was divided according to the survey method: household survey, health interview survey, and health behavior survey. The household survey (e.g., interview) focuses on one adult (19 years of age or older) in the household and surveys household type and incomes as well as the number of household members. The health interview investigated morbidity, medical use, activity restrictions, education and economic activities, and physical activities, and the health behavior survey (e.g., self-administered questionnaire) surveyed smoking, alcohol, mental health, safety awareness, and oral health [22,23]. Furthermore, the KNHANES has helped establish the health policy for national health development since 1988. Oh, Yang, Kim, and Kang [31] provided a result (e.g., Spearman correlation coefficient was 0.27) of correlation between the KNHANES of the IPAQ and the PA calculated by the accelerometer.

### 2.3. Research Variables

The variables of this study were chosen as the type of PA (e.g., walking, strength exercise, flexibility exercise) and HRQoL. In the development of reconstructing the selected research variables, a review of previous studies and discussions between researchers was analyzed. A variable as the type of PA was categorized by the aim of the study and the National Health and Nutrition Examination Guidelines [32]. The type of PA was walking, strength exercise, and flexibility exercise. For each PA type, there were questions (e.g., walking = “How many days did you walk at least 10 min at a time in the last week?”, strength exercise = “How many days did you perform strength exercises such as push-up, sit-up, dumbbell, iron bar, etc. in the last week?”, flexibility exercise = “How many days did you do flexibility exercises such as bare-hand, stretching exercise, etc. in the last week?”) and a 4-point scale (e.g., 1 = 5 days and over, 2 = 3–4 days, 3 = 1–2 days, 4 = Never). The research variables were shown in Table 2.

The variable HRQoL used Euro Quality of Life 5 Dimensions (EQ 5D index score) in evaluation, based on the standard proposed by the Korea Centers for Disease Control and Prevention to estimate the quality of life (QoL) of Koreans with depressive disorder. There are five areas: mobility (e.g., “I have no trouble walking,” “I have some trouble walking,” “I have to lie down all day”); self-care (e.g., “I have no trouble taking a bath or getting dressed,” “I have some trouble taking a bath or getting dressed alone,” “I cannot take a bath or get dressed alone”); usual activity (e.g., “I have no trouble doing my daily activities,” “I have some trouble doing my daily activities,” “I cannot do my daily activities”); pain/discomfort (e.g., “I have no pain or discomfort,” “I have some pain or discomfort,” “I have severe pain or discomfort”); anxiety/depression (e.g., “I am not anxious or depressed,” “I am somewhat anxious or depressed,” “I am very anxious or depressed”). The responses to each question are a 3-point scale with “1” indicating “no problem,” “2” indicating “some problems,” and “3” indicating “extreme problems.” Therefore, there are a total of 3^5^ = 243 different responses and levels of health that, according to QoL, can be measured. The data used in this study were accumulated by using the EQ-5D index score presented by the results of QoL in the KNHANES, considering the quality weights in the QOL. According to the Korean EQ-5D standard suggested by the Korea Centers for Disease Control and Prevention, a case in which all five areas are “1” is in complete health, and the EQ-5D value at this time is set to 1. If there is a response of “2” or “3,” EQ-5D = 1 − h, and “h” uses the weighting. M2 denotes the case where the response to the mobility is “2,” M3 is where the response to the mobility is “3,” and the rest of the responses are defined as well. The last N3 means there is a response of “3”.

### 2.4. Data Analysis

The aim of the study was to determine the effects of different types of PA (e.g., walking, strength exercise, flexibility exercise) on the HRQoL of Korean women with depression. The design of the complex sample suggested in the National Health and Nutrition Examination Guidelines [32] was used to accomplish the purpose of this study. The questionnaire area was allocated to the cluster after the data from 2007 to 2015 were vertically merged. The stratification variable was designated to the dispersion estimation layer. Furthermore, there were the integrated weights for analysis from 2007 to 2015, with the integration rate as 0.5/8.5 in 2007 and 1/8.5 from 2008 to 2015.

The characteristics of the participants were examined by the complex sample in frequency analysis. In addition, the complex sample general linear model was performed to identify the effects of different types of PA on the HRQoL of Korean women with depressive episodes. The significance level in this study was *p* < 0.05. To avoid bias in the dispersion estimator and the dispersion estimation after deleting missing data, the value of user-missing was shifted to valid values, and all elements with the missing data were contained for analysis. All statistical analyzes were conducted by the SPSS 18.0 statistical package.

## 3. Results

### Effects of Different Types of Physical Activity (e.g., Walking, Strength Exercise, Flexibility Exercise) on the HRQoL of Korean Women with Depressive Disorder

The complex sample general linear model was conducted to determine the effects of different types of PA (e.g., walking, strength exercise, flexibility exercise) on the score of the HRQoL of Korean women with depressive symptoms. In the case of Korean women with depressive episode, it was found that 30.7% could explain the HRQoL. According to the results of the frequency of participation by different types of PA (e.g., walking, strength exercise, flexibility exercise), it was found that there was a statistically significant difference in the group who participated for one to two days and three to four days from the group who did not participate in walking at all. In the flexibility exercise, there was a statistically significant difference in the group who participated in the three to four days from the group who did not participate at all. However, there was no statistically significant difference in strength exercise. The results of the effect of different types of PA on HRQoL of Korean women with depression are shown in Table 3.

## 4. Discussion

The hypothesis of the current study was that there are differences between the effects of different types of PA (e.g., walking, strength exercise, flexibility exercise) on the HRQoL of Korean women aged 19 or older with depressive disorder. The complex sample general linear model was performed to identify the aim of this study. Based on the results, Korean women with a depressive symptom who participated for one to two days and three to four days in walking had a higher level of HRQoL than the group who did not participate in walking at all. Furthermore, Korean women with a depressive disorder who participated for three to four days in flexibility exercise had a higher level of HRQoL than the group who did not participate in flexibility exercise at all. On the other hand, there was no statistically significant difference in strength exercise on the HRQoL of Korean women with a depressive disorder.

In the present study, PA, such as walking, contributed to improving the level of the HRQoL of Korean women with a depressive episode. These results were connected to outcomes by ESHGH et al. [33], who indicated that walking increases the scores of the physical aspects of QoL in mastectomy patients. Basen-Engquist et al. [34] stated that walking could significantly provide improvement of QoL for individuals with breast cancer. Many mastectomy patients develop a mental illness, such as depression after surgery, and their physical aspects of their QoL could be considerably improved after walking. In the study of Kang and Cho [35], it was found that the walking exercise group of middle-aged women has lower stress perception, anxiety, and depression in the HRQoL than the non-exercise group of middle-aged women. Walking exercise can be associated with daily activities; It does not need special costs, facilities, or instructions, and anyone can easily access PA by walking. In addition, there are many researchers that show that walking relieves depressive symptoms positively in the HRQoL. In this study, Korean women with a depressive disorder who participated for one to two days and three to four days in walking had higher scores of the HRQoL than the non-walking group. The Korea Ministry of Culture, Sports and Tourism [36] reported that around 45% of Korean women are doing moderate-intensity walking. However, the high-intensity (i.e., 5 days and over walking in a week in this study) of walking exercise or non-exercise cannot reduce and prevent depression and increase the level of the HRQoL based on results in this study [35]. Thus, the exercise intensity should be controlled to develop the HRQoL of Korean women with depressive symptoms.

In this study, there were no significant effects of strength exercise on the HRQoL of Korean women with depressive episodes. These findings are in line with earlier studies, in which Nilsen et al. [37] showed strength training did not affect the HRQoL. On the other hand, several studies reported the improvement of the HRQoL of participants through PA, including strength exercise [38,39,40]. Furthermore, the association between the level of grip strength and the HRQoL was greater in elderly women than in elderly men due to the effects of muscle strength loss [41]. However, these studies have determined the effects of other interventions (e.g., combined exercise program, different participants such as men, elderly people, and cancer patients) on the HRQoL. Based on the result (e.g., no significant effects of strength exercise on the HRQoL of Korean women with a depressive disorder) in this study, one factor must be considered that the participants of this study had depressive symptoms. Depression shows negative emotional states rather than positive states, which causes a loss of activity and enjoyment [42]. These emotional states have a crucial influence on mental health [43]. Some researchers [44] suggested that the QoL for patients can be affected more by depression than exercise. In addition, strength exercise can provide stress for participants because of confronting initial problems, such as learning exercise skills, exercise intensity, and exercise dosage (e.g., sets, repetitions) [45,46]. Hence, these factors would have a negative impact on the HRQoL of Korean women with depressive disorder, and whether strength exercise should be considered effective PA for the HRQoL of Korean women with a depressive disorder should be considered.

In the flexibility exercise, there was a statistically significant difference in the group who participated for three to four days from the group who did not participate at all. Esgalhado et al. [47] reported that flexibility exercises, such as stretching, helps to improve sub-factors (e.g., quality of rest, sleep, nutrition) of the HRQoL. It promotes psychological and physical stability, so people do not get stressed out. Stretching exercises can also have a beneficial effect on posture, reducing pain, improving balance, and increasing self-efficacy [48]. Similarly, pilates highlights respiratory control and muscle extending, which may have a positive mental and physical effect on the persons, making the HRQoL better reported [46]. However, there were no statistically significant differences between the group who participated for one to two days and five days and over and the group who did not participate at all.

These results were associated with findings by Stults-Kolehmainen and Sinha [49] that, which in the review paper on PA’s effects on stress, high-intensity PA might increase psychological stress and have negative effects, such as smoking, alcohol, and drug addiction. Consequently, the results presented in this study can appear differently according to individual characteristics in people with depressive disorder, so the type, intensity, duration, and time of PA (i.e., walking, flexibility) should be carefully considered [50].

## 5. Research Limitations

In the current study, there were some research limitations. First, the findings of this study might not be generalizable to all women who have a depressive episode in other countries, considering the participants aged 19 or above were recruited in South Korea. Furthermore, there were many studies related to South Korea; hence, the generalization of the outcomes in this study should be limited to Korean women aged 19 or older with a depressive disorder because of cultural differences. Providing evidence for generalization to other countries should be considered in future studies (e.g., including participants younger than 19 and participants and previous studies in different countries). The second limitation was that the results presented in this study do not provide data that can be compared to Korean women without depressive symptoms. This is because the numerical difference between the data of the two samples of Korean women with and without depression was so huge. Next research should be considered to provide data to compare between two groups in Korean women with and without depression to examine the efficacy of PA on the HRQoL of women with and without depression. The third restriction was that this study did not provide the data of events by the duration of each PA, which can affect errors in the rate of different types of PA (e.g., walking, strength exercise, flexibility exercise) by duration. Future research should present the data of events by the duration of each PA in women with a depressive disorder to meet the aim of the study and be clear to readers.

## 6. Conclusions

The aim of the current study was to identify the effects of different types of PA (e.g., walking, strength exercise, flexibility exercise) on the HRQoL of Korean women with a depressive disorder. There were the following conclusions: walking and flexibility exercise had significant effects on the HRQoL of Korean women with depressive episodes. However, strength exercise did not get significant effects on the HRQoL of Korean women with depressive disorder. Furthermore, there were strengths and applications of the study as we can see which PA is good for the HRQoL of Korean women with a depressive disorder. Future studies should be considered to determine the effects of different types of PA (e.g., except walking, strength exercise, flexibility exercise) on the HRQoL of women with a depressive disorder. The exercise intensity, exercise dosage, and exercise duration should be considered to identify the HRQoL of women with depressive symptoms in future studies.

## Figures and Tables

**Table 1:** Demographic characteristics of participants in the study.

Characteristics		Unweighted *N*	Weighted *N*	% (Weighted)
Age	19–29	74	66,517.570	9.1
	30–39	164	108,134.468	14.7
	40–49	187	142,869.839	19.5
	50–59	274	161,737.707	22.0
	60–69	331	134,858.558	18.4
	70≤	285	119,766.496	16.3
Economic activity	Employment	439	271,010.052	37.0
	Unemployment or economically inactive persons	871	460,540.748	63.0
Ownership of a house	0	472	307,171.841	41.9
	1	717	365,237.236	49.8
	2 more	124	608,080.363	8.3
Activity restriction	Yes	566	289,376.0	39.5
	No	747	443,782.3	60.5
Degree of stress recognition	Extremely	273	163,678.077	22.6
	Much	510	287,395.077	39.6
	Slightly	429	228,308.852	31.5
	Scarcely	91	45,478.838	6.3

**Table 2:** The variables of the study.

Variable		Question	Response Category	
			Original	Modified
Independent variable	Walking	How many days did you walk at least 10 min at a time in the last week?	1 = Never, 2 = 1 day, 3 = 2 days, 4 = 3 days, 5 = 4 days, 6 = 5 days, 7 = 6 days, 8 = everyday	1 = 5 days and over, 2 = 3–4 days, 3= 1–2 days, 4 = Never
	Strength exercise	How many days did you perform strength exercises, such as push-up, sit-up, dumbbell, iron bar, etc., in the last week?	1 = Never, 2 = 1 day, 3 = 2 days, 4 = 3 days, 5 = 4 days, 6 = 5 days and over	1 = 5 days and over, 2 = 3–4 days, 3= 1–2 days, 4 = Never
	Flexibility exercise	How many days did you do flexibility exercises, such as bare-hand, stretching exercise, etc., in the last week?	1 = Never, 2 = 1 day, 3 = 2 days, 4 = 3 days, 5 = 4 days, 6 = 5 days and over	1 = 5 days and over, 2 = 3–4 days, 3= 1–2 days, 4 = Never

**Table 3:** Results of the effects of different types of PA (e.g., walking, strength exercise, flexibility exercise) on HRQoL of Korean women with a depressive disorder.

Type of PA	Participation	Estimates of HRQoL	SE	*t*
Walking	5 days and over	0.014	0.016	0.877
	3–4 days	0.040	0.018	2.288 *
	1–2 days	0.049	0.019	2.645 **
	Never	0.000		
Strength Exercise	5 days and over	0.022	0.023	0.934
	3–4 days	−0.028	0.030	−0.930
	1–2 days	−0.016	0.023	−0.708
	Never	0.000		
Flexibility Exercise	5 days and over	0.027	0.015	1.799
	3–4 days	0.040	0.016	2.541 *
	1–2 days	0.007	0.015	0.502
	Never	0.000		
		R^2^ = 0.307 *p* < 0.000		

Note: * *p* < 0.05, ** *p* < 0.01. Adjusted variables: Age, economic activity, ownership of the house, activity restriction, and degree of stress recognition.

## Data Availability

Not applicable.

## References

[1] Munoz R.F., Beardslee W.R., Leykin Y. (2012). Major depression can be prevented. Am. Psychol..

[2] Center for Behavioral Health Statistics and Quality (2018). 2017 National Survey on Drug Use and Health Final Analytic File Codebook, Substance Abuse and Mental Health Service Administration.

[3] Evans J., Macrory I., Randall C. (2016). Measuring National Well-Being: Life in the UK.

[4] GBD 2017 Disease and Injury Incidence and Prevalence Collaborators (2018). Global, regional, and national incidence, prevalence, and years lived with disability for 354 diseases and injuries for 195 countries and territories, 1990–2017: A systematic analysis for the Global Burden of Disease Study 2017. Lancet.

[5] Cho M. (2011). The Epidemiological Survey of Psychiatric Illnesses in Korea: 2011.

[6] Andersson L., Sundström-Poromaa I., Bixo M., Wulff M., Bondestam K., Åström M. (2003). Point prevalence of psychiatric disorders during the second trimester of pregnancy: A population-based study. Am. J. Obstet. Gynecol..

[7] Yu K.Z. (2012). Factors influencing middle-aged women’s depression and anxiety. J. Korean Soc. Health Sci..

[8] Koo K.M., Kim K. (2020). Effects of physical activity on the stress and suicidal ideation in Korean adult women with depressive disorder. Int. J. Environ. Res. Public Health.

[9] Reeve B.B., Hays R.D., Bjorner J.B., Cook K.F., Crane P.K., Teresi J.A., Thissen D., Revicki D.A., Weiss D.J., Hambleton R.K. (2007). Psychometric evaluation and calibration of health-related quality of life item banks. Med. Care.

[10] Eljedi A., Mikolajczyk R.T., Kraemer A., Laaser U. (2006). Health-related quality of life in diabetic patients and controls without diabetes in refugee camps in the Gaza strip: A cross-sectional study. BMC Public Health.

[11] Lee W.J., Song K.H., Noh J.H., Choi Y.J., Jo M.W. (2012). Health-related quality of life using the EuroQol 5D questionnaire in Korean patients with Type 2 Diabetes. J. Korean Med..

[12] Anderson E., Shivakumar G. (2013). Effects of exercise and physical activity on anxiety. Front. Psychiatry.

[13] Rebar A.L., Stanton R., Geard D., Short C., Duncan M.J., Vandelanotte C. (2015). A meta-meta-analysis of the effect of physical activity on depression and anxiety in non-clinical adult populations. Health Psychol. Rev..

[14] Kim S.Y., Park J.H., Lee M.Y., Oh K.S., Shin S.W., Shin Y.C. (2019). Physical activity and the prevention of depression: A cohort study. Gen. Hosp. Psychiatry.

[15] Zhang X.C., Woud M., Becker E.S., Margraf J. (2018). Do health-related factors predict major depression? A longitudinal epidemiologic study. Clin. Psychol. Psychother..

[16] Mourady D., Richa S., Karam R., Papazian T., Hajj Moussa F., El Osta N., Kesrouani A., Azouri J., Jabbour H., Hajj A. (2017). Associations between quality of life, physical activity, worry, depression and insomnia: A cross-sectional designed study in healthy pregnant women. PLoS ONE.

[17] Pereira M.A., Rifas-Shiman S.L., Kleinman K.P., Rich-Edwards J.W., Peterson K.E., Gillman M.W. (2007). Predictors of change in physical activity during and after pregnancy: Project Viva. Am. J. Prev. Med..

[18] Robledo-Colonia A.F., Sandoval-Restrepo N., Mosquera-Valderrama Y.F., Escobar-Hurtado C., Ramírez-Vélez R. (2012). Aerobic exercise training during pregnancy reduces depressive symptoms in nulliparous women: A randomised trial. J. Physiother..

[19] Jung H., Kwak Y., Baek Y. (2011). The effects of aqua aerobics exercise on quality of life, depression, physical fitness, and blood lipid levels among climacteric women. J. Women’s Stud..

[20] Cho S. (2015). Effect of physical activity for school violence, suicide prevention. J. Korean Soc. Study Phys. Educ..

[21] Yi E., Lee S. (2009). The stress, depression, suicidal thoughts, and the buffering effect of leisure sports participation among the elderly in rural area. Korean J. Soc. Sport.

[22] Korea Ministry of Health and Welfare 2018 the Epidemiological Survey of Mental Disorders in Korea. http://www.bgnmh.go.kr/bgnmh/board/bgnmhHtmlView.jsp?menu_cd=BM_02_02_00_01&no=315.

[23] Korea Ministry of Health and Welfare (2011). 2011 the Epidemiological Survey of Mental Disorders in Korea.

[24] Yang J. (2010). Depression of women and remedies. Korean Acad. Soc. Womens Health.

[25] Lin Q.L., Kim H.K., Ann J.S. (2011). Relationship between depression and quality of life in elderly women living alone: The moderating and mediating effects of social support and social activity. J. Korean Gerontol. Soc..

[26] Chu I., Wu W., Lin I., Chang Y., Lin Y., Yang P. (2017). Effects of yoga on heart rate variability and depressive symptoms in women: A randomized controlled trial. J. Altern. Complement. Med..

[27] Bernard P., Ninot G., Bernard P.L., Picot M.C., Jaussent A., Tallon G., Blain H. (2015). Effects of a six-month walking intervention on depression in inactive post-menopausal women: A randomized controlled trial. Aging Ment. Health.

[28] LeCheminant J.D., Hinman T., Pratt K.B., Earl N., Bailey B.W., Thackeray R., Tucker L.A. (2014). Effect of resistance training on body composition, self-efficacy, depression, and activity in postpartum women. Scand. J. Med. Sci. Sports.

[29] IPAQ Research Committee Guidelines for Data Processing and Analysis of the International Physical Activity Questionnaire (IPAQ)-Short and Long Forms. https://sites.google.com/site/theipaq/scoring-protocol.

[30] Craig C.L., Marshall A.L., Sjöström M., Bauman A.E., Booth M.L., Ainsworth B.E., Pratt M., Ekelund U., Yngve A., Sallis J.F. (2003). International physical activity questionnaire: 12-country reliability and validity. Med. Sci. Sports Exerc..

[31] Oh J., Yang Y., Kim B., Kang J. (2007). Validity and reliability of Korean version of international physical activity questionnaire (IPAQ) short form. Korean J. Fam. Med..

[32] Korea Centers for Disease Control and Prevention (2016). The 6th National Health and Nutrition Examination Guidelines (2013–2015).

[33] Eshgh Z.M., Rahemi Z., Majd H.A., Hoviattalab S.K., Yaghamaei F. (2011). Effects of walking on quality of life of mastectomy patients at selected hospitals of Tehran. Iran. J. Nurs. Midwifery Res..

[34] Basen-Engquist K., Taylor C.L., Rosenblum C., Smith M.A., Shinn E.H., Greisinger A., Gregg X., Massey P., Valero V., Rivera E. (2006). Randomized pilot test of a lifestyle physical activity intervention for breast cancer survivors. Patient Educ. Couns..

[35] Kang I., Cho W. (2016). The influence on mental health status and health-related quality of life in middle-aged women by the regular walking exercise by based on the Korea National Health and Nutrition Examination Survey (KNHANES VI). J. Korean Soc. Wellness.

[36] Korea Ministry of Culture, Sports, and Tourism (2010). 2010 National Survey of Participation in Sports.

[37] Nilsen T.S., Raastad T., Skovlund E., Courneya K.S., Langberg C.W., Lilleby W., Fosså S.D., Thorsen L. (2015). Effects of strength training on body composition, physical functioning, and quality of life in prostate cancer patients during androgen deprivation therapy. Acta Oncol..

[38] Galvão D.A., Taaffe D.R., Spry N., Joseph D., Newton R.U. (2010). Combined resistance and aerobic exercise program reverses muscle loss in men undergoing androgen suppression therapy for prostate cancer without bone metastases: A randomized controlled trial. J. Clin. Oncol..

[39] Segal R.J., Reid R.D., Courneya K.S., Malone S.C., Parliament M.B., Scott C.G., Venner P.M., Quinney H.A., Jones L.W., Slovinec D’Angelo M.E. (2003). Resistance exercise in men receiving androgen deprivation therapy for prostate cancer. J. Clin. Oncol..

[40] Cormie P., Galvao D.A., Spry N., Joseph D., Chee R., Taaffe D.R., Chambers S.K., Newton R.U. (2015). Can supervised exercise prevent treatment toxicity in prostate cancer patients initiating androgen deprivation therapy: A randomised controlled trial. Br. J. Urol. Int..

[41] Sayer A.A., Syddall H.E., Martin H.J., Dennison E.M., Roberts H.C., Cooper C. (2006). Is grip strength associated with health-related quality of life? Findings from the Hertfordshire Cohort Study. Age Ageing.

[42] Krumholz H.M., Butler J., Miller J., Vaccarino V., Williams C.S., Mendes de Leon C.F., Seeman T.E., Kasl S.V., Berkman L.F. (1998). Prognostic importance of emotional support for elderly patients hospitalized with heart failure. Circulation.

[43] Rezvan A.F., Srimathi N.L. (2017). Assessment of stress among Iranian students and its relationship with depression and anxiety. Indian J. Health Wellbeing.

[44] Müller J., Hess J., Hager A. (2012). Minor symptoms of depression in patients with congenital heart disease have a larger impact on quality of life than limited exercise capacity. Int. J. Cardiol..

[45] Wermelinger Âvila M.P., Caputo Correa J., Lamas Granero Lucchetti A., Lucchetti G. (2018). The role of physical activity in the association between resilience and mental health in older adults. J. Aging Phys. Act..

[46] Kofotolis N., Kellis E., Vlachopoulos S.P., Gouitas I., Theodorakis Y. (2016). Effects of Pilates and trunk strengthening exercises on health-related quality of life in women with chronic low back pain. J. Back Musculoskelet. Rehabil..

[47] Esgalhado M., Stockler-Pinto M.B., de França Cardozo L.M., Costa C., Barboza J.E., Mafra D. (2015). Effect of acute intradialytic strength physical exercise on oxidative stress and inflammatory responses in hemodialysis patients. Kidney Res. Clin. Pract..

[48] Roddy E., Zhang W., Doherty M. (2005). Aerobic walking or strengthening exercise for osteoarthritis of the knee? A systematic review. Ann. Rheamatic Dis..

[49] Stults-Kolehmainen M.A., Sinha R. (2014). The effects of stress on physical activity and exercise. Sports Med..

[50] Ströhle A. (2009). Physical activity, exercise, depression, and anxiety disorders. J. Neural Transm..

